# Radiation Oncology – Towards a mission‐oriented approach to cancer

**DOI:** 10.1002/1878-0261.12730

**Published:** 2020-07-02

**Authors:** Michael Baumann, Carol Bacchus

**Affiliations:** ^1^ German Cancer Research Center (DKFZ) Heidelberg Germany

Driven by the Boards of the European Academy of Cancer Sciences, Cancer Core Europe, and Cancer Prevention Europe, this thematic issue on radiation oncology is part of a series of articles addressed to both the scientific community and the decision‐makers working on a mission‐oriented approach to cancer in the context of Horizon Europe (Eggermont *et al*., 2019; Wild *et al*., 2019). The first issue of the series, entitled: Boosting the Social Impact of Innovative Cancer Research – Towards a Mission‐oriented Approach to Cancer, was published in *Molecular Oncology* in March 2019.

For more than a century, radiation oncology has established itself as a mainstay of cancer treatment. As cancer continues to pose a major health risk and the global incidence rate is expected to almost double in the next two decades, the importance of radiation therapy will continue to grow. Radiation oncology will be shaped by oncoming advances in medicine, including early and lower‐cost detection methods, integrated multidisciplinary approaches, personalized medical strategies, and cancer prevention. In addition, radiation oncology will be influenced by future societal changes, including the establishment of more efficient, value‐based healthcare systems, and, importantly, extended European and global cooperation in research and care for the fight against disease.

The importance of a common European mission is evident in view of the current enormous disparity regarding patient access to state‐of‐the‐art radiotherapy from one European country to the other (Lievens *et al*., 2020). This disparity, which is alarming given the indispensability of radiotherapy in cancer treatment, can be attributed to several factors:

1. The establishment of a radiotherapy department needs considerable upfront investment in state‐of‐the‐art diagnostic and therapeutic technologies and devices. Nevertheless, as these devices can be used for many years, radiotherapy enables cost‐efficient treatment of thousands of patients.
2. Radiotherapy needs to be administered by well‐trained personnel and by multiprofessional teams. But, Europe does not have enough well‐trained staff and will need national overarching efforts to train a sufficient number of experts in the coming years. These expert teams should consist at least of radiation oncologists, medical physicists, and radiation therapy technologists. In addition, radiation biologists, data scientists, engineers, and experts from many other fields are of fundamental importance for the further development of radiation oncology.
3. Radiotherapy requires a certain degree of centralization to ensure that a critical mass of equipment and personell are available for delivering all of radiotherapy required by current and future cancer treatment regiments. Consequently, radiotherapy departments cannot be established in all medical centers and sites within a country. This can cause problems for decision‐makers as to selecting locations for radiotherapy centers.
4. Radiotherapy is only one component of cancer treatment, as it is always combined with other diagnostic and therapeutic disciplines in a multidisciplinary framework. Radiotherapy departments are therefore best located within multidisciplinary cancer treatment units, such as comprehensive cancer centers.
5. If decentralized radiotherapy units are needed to provide access for all citizens nationwide, these units can, for example, be connected to larger network centers. In general, decentralized services closer to the patients’ homes are more beneficial and practical for palliative care, or if tumors require well‐defined and frequently applied standard treatments. More specialized treatment should ideally be provided by larger departments. Last but not least, modern telemedicine connectivity and reliable and ubiquitous patient referral systems are needed to ensure that even patients living in more remote areas have access to all treatments offered.

Finally, going beyond patient care, Europe also needs innovation. Europe has a strong track record not only in biological research and drug development, but particularly also in medical engineering and medical technologies that form the backbone of modern radiotherapy. As the driving ideas behind new generations of technology often stem from close interactions among the clinical care sector, academic research, and industry, investment into radiation oncology will also boost European research and economies.

This thematic issue in *Molecular Oncology* discusses central questions of the future direction and development of radiotherapy in Europe. The cancer mission must take into account the needs of tomorrow’s aging population, as well as the nationwide diversity of systems in Europe. This is only possible if all research areas are brought together in a symbiotic approach to cure and slow down tumor growth and combat comorbidities.

We would like to thank the authors of this special issue for providing the data and for laying the foundation for further developments in radiation oncology, covering a broad range of topics from innovative, technology‐, and data‐driven approaches to translational medicine and personalized strategies to cancer prevention and patient care in radiation oncology (Lievens *et al*., 2020; Wild *et al*., 2019; Lieverse *et al*., 2020; Vogelius *et al*., 2020; Grau *et al*., 2020; Leech *et al*., 2020; Krause *et al*., 2020; Fiorino *et al*., 2020; Mondini *et al*., 2020; Valentini *et al*., 2020; Baumann *et al*., 2020; Barazzuol *et al*., 2020; Vincent *et al*., 2020).
